# Supplementary material for: Amiselimod (MT-1303), a novel sphingosine 1-phosphate receptor-1 functional antagonist, inhibits progress of chronic colitis induced by transfer of CD4+CD45RBhigh T cells
Source: PLoS One. 2019 Dec 5;14(12):e0226154. doi: 10.1371/journal.pone.0226154 (PMC6894856; doi:10.1371/journal.pone.0226154)
Supplement: Attachment — Submitted filename: Response to Reviewers _shimano.doc [file pone.0226154.s006.doc]

**Responses to the comments of Reviewer 1**

Comments to the Author

The experiment model was almost enough to support MT1303 suppress the colitis. However, I would like to ask some questions below.

1. First of all, authors need to add the physiological condition. After orally administrated MT1303 to the B6 or Balbc mice, authors need to show the number of Th1/Th17/Treg cells in LP, mesenteric lymph node. Authors also need to add the cell number under the long term administration at least 28days. Show the macro picture of colon and mesenteric lymph node.

*Response: Unfortunately, we did not evaluate the changes in the number of Th1, Th17 and Treg cells when MT -1303 was administered to normal mice. It has been reported that administration of FTY 720, the first S1P1 receptor modulator, to C57BL/6 mice for 21 days caused a transient increase in the number of CD4 and CD8 T cells in the lymph nodes over the first 2 days, followed by a gradual decrease to levels slightly lower than those at day 0 (Margaret A., Eur. J. Immunol., 2005;35:3570-80). In long-term toxicity studies in mice, rats and monkeys, no particular changes were observed in the pathological examination of intestinal tissue.*

2. Second, in figure3 and 4 authors showed the MT1303 antagonist ameliorated colitis. I would ask the number of Tregs in these experiments, because Tregs are also essential to protect experimental colitis model (Mottet C. JI 2003.).

*Response: Thank you for this comment. In this study, we focused on Th1 and Th 17 cells because we transferred naïve CD4^+^CD45RB^high^ T cells without Treg cells. However, in a colitis model in which CD4^+^CD45RB^high^ T cells were transfected into Rag1-/- mice, it was reported that Foxp3^+^CD4^+^ Treg cells start to appear at 4 weeks and gradually increase until 8 weeks (Chen W., Cell Mol Immunol., 2017;14:1013-6). Therefore, it is very interesting to clarify how Treg cells are affected by MT-1303 treatment in the colitis model. We add the following sentence in Discussions.* *“In addition to Th1 and Th17 cells, CD4+FoxP3+ regulatory T cells (Tregs) also play an important role in development of colitis [26], and in the future study, it is necessary to clarify how the administration of MT -1303 affects the number or frequencies of Tregs in LP and mesenteric lymph nodes in this model.”*

3. Finally, I would ask whether MT1303 cure the colitis. To solve the problem, I would ask colitis is ameliorated when MT1303 antagonist is administrated from day 1 to day 14 and observe until day 35 in experimental colitis model.

*Response:* *When MT-1303 at a sufficient dose is continuously administered to the mice from the day of cell transfer, it is not expected to develop colitis. However, if MT-1303 administration is discontinued, there is a possibility that colitis will develop gradually or not develop. We do not have a clear answer at this time.*

**Responses to the comments of Reviewer 2**

In this manuscript, Shimano et al demonstrated a novel S1P1R antagonist MT-1303 effectively inhibited the development of colitis to prevent infiltrating pathogenic Th1 and Th17 cells into colonic lamina propria. Although it is quite interesting, I have several comments as below.

1. Other than Th1 and Th17 cells, Foxp3+ regulatory T cells (Tregs) are also induced in this colitis model. How Tregs are affected by MT-1303 treatment?

*Response: Thank you for this comment. We have not evaluated effect of MT-1303 on Treg cells in the colitis model. In our preliminary study, it has been confirmed FTY720 administration decreases Treg cell counts, but increased ratio of Treg cells in CD4^+^ T cells in DLNs of EAE mice. As you commented, because Treg cells are also induced in the colitis model, it is very interesting to clarify how Treg cells are affected by MT-1303 treatment. We add the following sentence in Discussions. “In addition to Th1 and Th17 cells, CD4^+^FoxP3^+^ regulatory T cells (Tregs) also play an important role in development of colitis [26], and in the future study, it is necessary to clarify how the administration of MT -1303 affects the number or frequencies of Tregs in LP and mesenteric lymph nodes in this model.”*

2. A previous publication of the authors (Chiba K et al, Int Immunopharmacol (2003)) showed that the ratio of both Th1 and Th17 in local site (spinal cord) was lower but that in lymph node was higher by FTY720 treatment in EAE model. However, in the colitis model, it seems curious because indeed absolute number of infiltrating T cells was lower, no significant change of the ratio was observed in the intestine. Please explain this discrepancy.

*Response: We think that differences of T cell stimulation in two studies may influence the results. In EAE study, mice were immunized with PLP, and PLP-specific Th1 and Th17 cells were detected in spinal cords and LNs. Meanwhile, in the present study, T cells from LP were stimulated with PMA and ionomycin, and then non-specific Th1 and Th 17 cells were measured. When we detect colitogenic Th1 and Th17 cells, which expand in MLN, infiltrated into LP and associated with the pathophysiology of colitis, in the colitis model, it is expected that ratios of Th1 and Th 17 cells were decreased in LP and increased in MLNs by MT-1303 administration*

3. It is well known that IL-10 has a regulatory role of immune responses of the intestine. The authors should check whether IL-10 expression in T cells are affected by MT-1303 treatment.

*Response: Unfortunately, the effect on IL -10 production has not been evaluated in this study. In the similar model, administration of another S1P1 receptor modulator (etrasimod) has been reported to increase IL -10 mRNA level and decrease IL-17A mRNA level in the colon (AI-Shmma H., J. Pharmacol. Exp. Ther., 2019;369:311-7).*
