# Supplementary material for: Amiselimod (MT-1303), a novel sphingosine 1-phosphate receptor-1 functional antagonist, inhibits progress of chronic colitis induced by transfer of CD4+CD45RBhigh T cells
Source: PLoS One. 2019 Dec 5;14(12):e0226154. doi: 10.1371/journal.pone.0226154 (PMC6894856; doi:10.1371/journal.pone.0226154)
Supplement: Attachment — Submitted filename: Response letter3_shimano.doc [file pone.0226154.s007.doc]

Professor Akihiko Yoshimura

Academic Editor

PLOS ONE

19 November 2019

Dear Professor Yoshimura,

Re: Resubmission of manuscript PONE-D-19-23451R1

Please find attached a revised version of our manuscript “Amiselimod (MT-1303), a novel sphingosine 1-phosphate receptor-1 functional antagonist, inhibits progress of chronic colitis induced by transfer of CD4^+^CD45RB^high^ T cells,” which we would like to resubmit for consideration for publication as a Research Paper in the *PLOS ONE*.

According to your comments, we have conducted an experiment to evaluate the effect of MT-1303 on Tregs in normal mice. In the following pages, our responses to your comments are presented including the results of experiments.

Revisions in the text are shown using yellow highlight. Please let us know, if there are any parts that do not meet your requests.

We hope that the revisions in the manuscript and our accompanying responses will be sufficient to make our manuscript now suitable for publication in *PLOS ONE.*

We look forward to hearing from you at your earliest convenience.

Yours sincerely,

Kunio Sugahara, Ph.D.

Sohyaku Innovative Research Division

Mitsubishi Tanabe Pharma Corporation

1000 Kamoshida-cho, Aoba-ku, Yokohama 227-0033, Japan

E-mail: Sugahara.Kunio@me.mt-pharma.co.jp

Tel.: +81-45-963-3057

Fax: +81-45-963-4641

**Additional Editor Comments (if provided):**

Both reviewers asked the effect of MT-1303 on Treg cells in the colitis model. However authors did not provide any evidence concerning this point. Unfortunately, no additional experimental data was provided. It is very hard to accept this paper without any additional information. Please perform at least one experiment.

*Response: Thank you for the comment. We have assessed the effect of MT-1303 on the number of Tregs in peripheral blood, mesenteric lymph nodes (MLNs) and spleen in normal C57BL/6 mice.* *After oral administration of MT-1303 at 1 mg/kg once daily for 7 days, blood, MLNs and spleen were collected from C57BL/6 mice* *under isoflurane anesthesia, and the number of CD4^+^CD25^+^FoxP3^+^Tregs and CD4^+^T cells were determined by flow cytometry. MT-1303 treatment drastically decreased the number of Tregs and CD4+T cells in peripheral blood (Figs 1B and 1A). On the other hand, administration of MT-1303 significantly increased the number of Tregs in MLNs (Fig 1D) and the percentage of Tregs in CD4+T cells in MLNs and spleen (Figs 1F and 1J).*

*It has been confirmed that MT-1303 affects Treg trafficking and increases both the number and percentage of Tregs in MLNs in normal mice. We have added the following sentences to the Discussion (lines 391-399).*

*CD4^+^FoxP3^+^ regulatory T cells (Tregs), in addition to Th1 and Th17 cells, also play an important role in development of colitis [26]. When MT-1303 was administered to C57BL/6 mice daily for 7 days, the number of Tregs (CD4^+^CD25^+^FoxP3^+^) as well as CD4^+^T cells in peripheral blood was decreased dramatically, although both the number of Tregs and the proportion of Tregs in CD4^+^T cells were increased in MLNs compared to vehicle-treated control mice (unpublished data). Since MT-1303 treatment influences Treg trafficking in normal mice, in the future study, it is important to clarify how the administration of MT -1303 affects the number or frequencies of Tregs in colon lamina propria and MLNs in this model.*

**Fig 1. Effect of MT-1303 on the number of Tregs in C57BL/6 mice.**　MT-1303 (1 mg/kg) or vehicle was orally administered to C57BL/6 mice daily for 7 days. The number of CD4^+^ T cells (A, C, G) and Tregs (CD4^+^CD25^+^FoxP3^+^) (B, D, H) in peripheral blood (A, B), MLNs (C, D) and spleen (G, H) were measured using flow cytometry on the day after the final administration, and the percentages of CD4^+^T cells (E, I) among T cells and of Tregs (F, J) among CD4+T cells in MLNs (E, F) and spleen (I–J) were calculated. Data points are individual mice and each bar represents the mean ± S.E.M. (n=4). Statistical differences were determined using Student’s *t*-test by comparing with the vehicle-treated control group (**p* < 0.05, ***p* < 0.01).
